# Mycoplasma mycoides, from "mycoides Small Colony" to "capri". A microevolutionary perspective

**DOI:** 10.1186/1471-2164-12-114

**Published:** 2011-02-16

**Authors:** Francois Thiaucourt, Lucia Manso-Silvan, Woubit Salah, Valérie Barbe, Benoit Vacherie, Daniel Jacob, Marc Breton, Virginie Dupuy, Anne Marie Lomenech, Alain Blanchard, Pascal Sirand-Pugnet

**Affiliations:** 1 CIRAD, UMR CMAEE, F-34398 Montpellier, France; 2 CEA/IG/Genoscope, Evry, France; 3 Univ. Bordeaux, CBiB, Bordeaux, 33076, France and Laboratoire Bordelais de Recherche en Informatique, UMR CNRS 5800, Talence, 33405, France; 4 INRA, UMR 1332, Villenave d'Ornon, 33883, France; 5 Univ. Bordeaux, UMR 1332, Villenave d'Ornon, 33883, France; 6 Univ. Bordeaux, pôle protéomique, 33076 Bordeaux, France; 7 College of Veterinary Medicine, Tuskegee University, AL 36088, USA

## Abstract

**Background:**

The *Mycoplasma mycoides *cluster consists of five species or subspecies that are ruminant pathogens. One subspecies, *Mycoplasma mycoides *subspecies *mycoides *Small Colony (MmmSC), is the causative agent of contagious bovine pleuropneumonia. Its very close relative, *Mycoplasma mycoides *subsp. *capri *(Mmc), is a more ubiquitous pathogen in small ruminants causing mastitis, arthritis, keratitis, pneumonia and septicaemia and is also found as saprophyte in the ear canal. To understand the genetics underlying these phenotypic differences, we compared the MmmSC PG1 type strain genome, which was already available, with the genome of an Mmc field strain (95010) that was sequenced in this study. We also compared the 95010 genome with the recently published genome of another Mmc strain (GM12) to evaluate Mmc strain diversity.

**Results:**

The MmmSC PG1 genome is 1,212 kbp and that of Mmc 95010 is ca. 58 kbp shorter. Most of the sequences present in PG1 but not 95010 are highly repeated Insertion Sequences (three types of IS) and large duplicated DNA fragments. The 95010 genome contains five types of IS, present in fewer copies than in PG1, and two copies of an integrative conjugative element. These mobile genetic elements have played a key role in genome plasticity, leading to inversions of large DNA fragments. Comparison of the two genomes suggested a marked decay of the PG1 genome that seems to be correlated with a greater number of IS. The repertoire of gene families encoding surface proteins is smaller in PG1. Several genes involved in polysaccharide metabolism and protein degradation are also absent from, or degraded in, PG1.

**Conclusions:**

The genome of MmmSC PG1 is larger than that of Mmc 95010, its very close relative, but has less coding capacity. This is the result of large genetic rearrangements due to mobile elements that have also led to marked gene decay. This is consistent with a non-adaptative genomic complexity theory, allowing duplications or pseudogenes to be maintained in the absence of adaptive selection that would lead to purifying selection and genome streamlining over longer evolutionary times. These findings also suggest that MmmSC only recently adapted to its bovine host.

## Background

Now that rinderpest has been eradicated, *M. mycoides *subsp. *mycoides *Small Colony (MmmSC), the aetiologic agent of contagious bovine pleuropneumonia (CBPP), is considered to be the most important threat to cattle farming in affected countries. Although CBPP has been eradicated from most continents, it persists in Africa. The disease is notifiable to the world organisation for animal health (OIE) and, following notification, export of live cattle to countries free of CBPP is forbidden. Programmes for the control of CBPP involve vaccination, but eradication is based solely on slaughter of affected herds and strict control of animal movements. Because of its economic importance, CBPP has received much attention especially when it affected industrialized countries. This was the case in the 1960's when Australia started an eradication programme and in the 80's and 90's when Europe suffered a re-emergence of the disease [1,2]. Identification and diagnostic methods have been improved. This task was difficult as MmmSC belongs to a complex of species, the so-called "*M. mycoides *cluster". This cluster consists of five closely related mycoplasmas that are referred to as: *M. mycoides *subsp. *mycoides *Small Colony (MmmSC), *M. mycoides *subsp. *capri *(Mmc), *M. capricolum *subsp. *capricolum *(Mcc), *M. capricolum *subsp. *capripneumoniae *(Mccp), and *Mycoplasma leachii* (Ml), the last being a group of strains that had remained unassigned until recently when a modification of the cluster taxonomy was proposed based on both phenotypic and recent phylogenetic studies [3,4]. The designation *Mycoplasma mycoides *subsp. *mycoides *Large Colony (MmmLC) was discarded and the corresponding "LC" isolates are now considered to be an additional serovar of Mmc [4]. All members of the *M. mycoides *cluster share phenotypic and genetic traits. One very close relative of MmmSC is Mmc, and indeed, the reference growth inhibition test using rabbit hyperimmune serum does not allow differentiation between these two subspecies. However, it is important to be able to identify MmmSC and Mmc without ambiguity because the two organisms differ greatly in terms of pathogenicity, geographical distribution and quarantine regulations. Unlike MmmSC, Mmc affects mostly small ruminants where it can induce a syndrome called "MAKePS" with lesions including mastitis, arthritis, kerato-conjonctivitis, pneumonia and septicaemia [5]. It can also be found in the ear canal of asymptomatic animals [6]. Mmc strains are found world-wide, especially where goats are raised. Before 1994, the distinction of the two subspecies *in vitro *was difficult and findings were sometimes ambiguous as few tests were sufficiently discriminatory (Table 1). Analysis of cellular proteins by one-dimensional SDS PAGE showed that this approach could be used to distinguish MmmSC from Mmc [7]. Specific detection methods were then developed based on PCR technology and the empirical search for specific restriction sites in the amplified fragment or with PCR primers allowing a specific amplification [8,9]. Finally, MmmSC specific monoclonal antibodies were obtained and, more recently, specific real-time PCR methods have also been validated [10-13].

**Table 1:** Phenototypic characteristics differentiating *M. mycoides *subsp. *mycoide*s SC (MmmSC) and *capri *(Mmc)

*Characteristics*	*MmmSC*	*Mmc*
Mean colony size after 137 h at 37°C	0.99 mm	2.26 mm
Liquefaction of inspissated serum	weak	+
Casein digestion	-	+
Sorbitol fermentation	-	+
Presence of ornithine transcarbamylase	-	+
Presence of alpha-glucosidase	-	+
Thermal stability at 45°C	sensitive	resistant
Digestion of DNA by MboI	+	-
DpnI	-	+
*Substrate oxidation (12-50 μM)*		
Maltose	-	+
Trehalose	-	V
Mannose	-	+
Glucosamine	-	+

List of phenotypic differences that allow the differentiation of *M. mycoides *subsp. *mycoides *small colony (SC) and *M. mycoides *subsp. *capri in vitro*. Adapted from [39,68-70].

The two subspecies have been considered to be very close relatives that could be distinguished only by minute differences *in vitro *in spite of their marked differences of physiology *in vivo*. Recent advances in sequencing and bioinformatics allow the comparison of whole bacterial genomes. The complete genome sequence of the MmmSC reference strain PG1 was made public in 2004 [14]. Here, we report the availability of the complete genomic sequence of an Mmc strain and compare it with that of the MmmSC PG1 strain. We describe differences in chromosome organization, gene repertoire, sequence polymorphism, and consider possible links between these differences and the physiology of the two bacteria. In addition, the recent publication of the genome sequence of strain GM12 (Mmc) allowed a genome-wide comparison for these two strains and an evaluation of intra-species polymorphism.

## Results

### General genome features

The Mmc 95010 genome consists of a circular chromosome of 1,153,998 bp (EMBL/GenBank accession number Q377874) and of a plasmid of 1,840 bp (pMmc-95010, GenBank accession number FQ790215). The chromosome has a G+C content of only 23.8 mole%. Genome annotation revealed 924 putative CDS, two Integrative Conjugative Elements (ICE), each being on average 30 kbp long and including 18 CDS, and 24 recognized copies of Insertion Sequences (IS) belonging to five different IS types. Two sets of rRNA genes and 30 tRNAs can be predicted. Putative genes corresponding to the tmRNA and the RNA component of the RNase P were also found, as in other mycoplasmas.

### Overview of genome structure and plasticity

**Figure 1:**
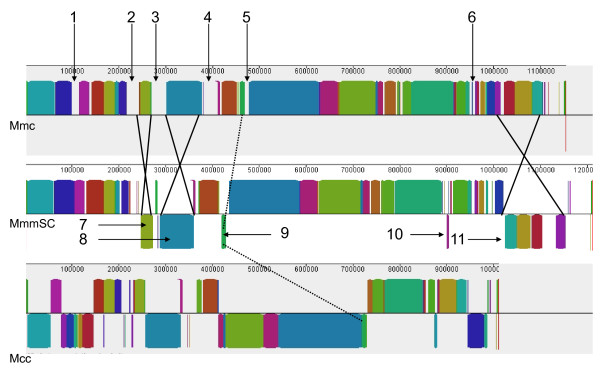
**Comparative genome structure of strains 95010 (Mmc), PG1 (MmmSC) and California kid (Mcc)**. The graph represents an alignment of the colinear blocks, identified by MAUVE, that are conserved in three closely related genomes: Mmc 95010; MmmSC PG1; Mcc California kid. Numbers 1 to 6 refer to large DNA fragments of the Mmc 95010 genome with apparently no homology in the other two genomes. Numbers 7 to 11 indicate DNA inversions (blocks below the central line) that distinguish Mmc from MmmSC. Comparison with the strain California kid genome allows identification of the genome in which these inversions have taken place. For blocks 7, 9, 10 and 11, the inversion took place in the MmmSC PG1 genome, whereas inversion 8 took place in the Mmc 95010 genome. The higher number of block rearrangements in the Mcc genome is in agreement with its taxonomic position and greater evolutionary distance from Mmc, as compared with MmmSC.

The MAUVE alignment of Mmc 95010, MmmSC PG1, and the slightly more distant California kid (Mcc) genome sequences allowed the identification of 62 Locally Collinear Blocks (LCB) that were interspaced by Mmc-specific DNA stretches of various lengths (Figure 1). Six of these Mmc-specific stretches merit special attention.

The first contains ten CDS (MLC_0740 to MLC_0840), six of which constitute a predicted lipoprotein (lpp) family for which the best similarity scores were found with *M. agalactiae *or Mcc, whereas no homologues were found in MmmSC. This gene family was previously described as a candidate for an Horizontal Gene Transfer (HGT) between *M. agalactiae *and the M. mycoides cluster [15,16]. The absence of homologues in MmmSC may be the consequence of a deletion from this pathogen after the HGT event.

The second is a stretch of 12 CDS (MLC-1730-1830) forming a maltodextrin/maltose gene cluster. Most of these CDS exhibit substantial similarity with genes in distantly related genera, such as *Listeria *or *Bacillus*, again suggestive of acquisition by HGT. Interestingly, the last four genes (MLC_1810 to 1840) had homologues in Mcc, an indication that Mcc may have previous contained, and then lost, the first eight CDS of the cluster.

The third (MLC_2080 to MLC_2280) and fourth (MLC_2890 to MLC_3080) specific DNA stretches correspond to two copies of the Integrative Conjugative Element (ICE) (see below) that are 30 and 28 kbp long, respectively.

The fifth specific DNA stretch contains a pseudogene and six CDS (MLC_3580 to MLC_3640). The second and third CDS were similar to two ICE CDS (MLC_3590 and MLC_3600) with the last four CDS being identified as IS*1296 *copies. Therefore, this DNA stretch appears to be the remains of an ICE copy after a partial deletion.

Finally, the sixth specific DNA stretch contains five CDS (MLC_7610 to MLC_7650) with four of them showing either no similarity to known sequences in any organism or showing similarity with sequences in non-mollicute organisms (*Treponema denticola, Trichomonas vaginalis, Finegoldia magna*), suggesting again a possible indication of HGT. MLC_7610 and MLC_7620 show similarities with viral A-type inclusion protein, consistent with this possibility.

The MAUVE genome alignment clearly indicated inversions of large DNA fragments including one or more collinear blocks. Only five of such events were identified between Mmc and MmmSC (Figure 1 LCB 7 to 11) and nine with Mcc, consistent with Mmc and MmmSC being more closely related to each other than to Mcc. The initial orientation of these LCB could be deduced for those that were colinear and with a similar orientation in two of the three genomes. For four of the five (LCB 7, 9, 10, 11), the DNA inversion occurred in the MmmSC genome with the inverted LCBs being flanked by IS copies (IS*1634 *or IS*1296*). The exception is LCB 8, which is flanked by two ICE copies in opposite direction in the Mmc-95010 genome, an indication that this inversion occurred during the duplication and insertion of the first ICE copy (see below)

### Mobile elements: Integrative Conjugative Elements

**Figure 2:**
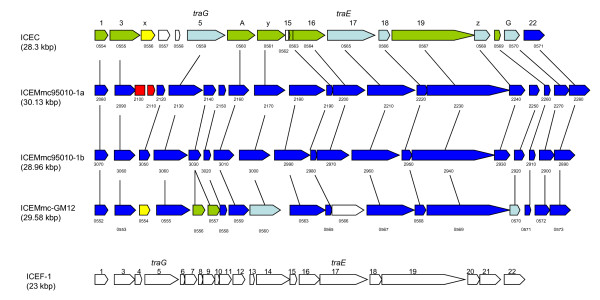
**Comparison of the two ICEs found in (95010) with those in Mcc (California kid) and *M. fermentans***. Schematic representation of ICEs found in Mcc (top) and *M*. *fermentans *(bottom) with the two ICE copies found in the Mmc 95010 genome and the single copy identified in strain Mmc GM12. The numbering above refers to that of the original publications describing these ICEs [16,26], and numbering below the ICEs refers to CDS numbers in the respective genomes. The CDS are coloured according to the similarity results from BLASTP with the ICEMmc95010-1a CDS (dark blue, #90%; light blue, 89-70%; green, 69-50%; no colour, < 50%). The two ICE copies in 95010 differ by two CDS (in red) which may have been deleted from the "1b" copy (or inserted into the "1a" copy). The ICE copy in GM12 is very similar as 13 CDS exhibit more than 90% similarity with ICEMmc95010 -1a CDS. However, some CDS are only present in GM12 (MMCAP2_0566), another is duplicated (MMCAP2_0556 and 0557) and another (MMCAP2_0554), in yellow, is similar to MCAP_0556. The greatest similarities were found within the TraE and TraG proteins and for the 2 CDS at the extremities. These terminal CDS may be joined and form a single CDS in the circular form of the ICE.

The two ICE copies identified in the 95010 genome were not found in the other available genomes of the *M. mycoides *cluster, including Mmc GM12. This genome contains an ICE but it is located elsewhere on the chromosome. This suggests that these mobile elements were recently inserted into the 95010 genome. The two ICE copies differ slightly in size (ICEMmc95010-1a is 30,136 bp-long, with 21 CDS, and ICEMmc95010-1b is 28,965 bp-long, with 19 CDS) but are otherwise almost identical (Figure 2). The CDS are very similar to those of the ICE found in Mcc (six of the 18 Mcc CDS exhibit more than 70% similarity to ICEMmc95010-1a CDS). Four CDS seemed to be more conserved among mycoplasmas: CDS 1 and 22, at the extremities of the ICE but contiguous in the circular form, and CDS 5 and 17, which encode TraG and TraE, respectively. For CDS 5 and 17, the highest similarity was found with homologues in Mmc strain GM12 (98%), Mcc California kid (80%), and partial sequences of *M. agalactiae *PG2 (80%). They were more distantly related to an *M*. *fermentans *CDS (50%).

**Figure 3:**
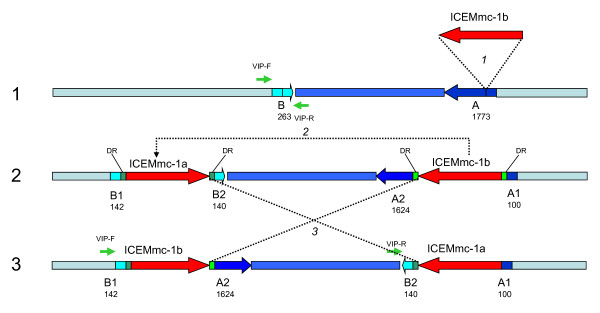
**Putative model of the insertion and duplication of an ICE copy in Mmc 95010 genome**. This figure describes the events that may have led to the presence of two ICE copies in the Mmc 95010 genome. A first ICE copy may have been acquired from a donor cell by conjugation, with the subsequent insertion in CDS A (MLC_2290). This insertion led to the disruption of CDS A resulting in fragments (A1 and A2) and the creation of a small Direct Repeat at the extremities of the ICE (DR). The ICE duplicated and inserted elsewhere in the genome, disrupting another CDS (CDS B, MLC_2070) and creating two additional direct repeats. Recombination between the two ICE copies then lead to the inversion of the DNA fragment located between them. The presence and the site of insertion of ICE copies in Mmc is strain specific. This was shown by PCR amplification with two primers chosen on either side of the ICE Mmc95010-1a integration site (primers VIP-F and VIP-R). In four Mmc strains, this PCR amplified the expected fragment, an indication that there was no ICE copy inserted at this site (see table 2).

The two Mmc 95010 ICE copies are in opposite orientation and flank an 80.9 kbp-long DNA fragment which is inverted, in relation to the most closely related genomes (Mmc strain GM12, MmmSC and Mcc). Examination of the direct repeats at the insertion sites of the ICE clearly showed that a recombination had occurred between them, resulting in the observed chromosomal inversion. It is therefore likely that the organization of this region is the result of successive events including the initial integration of an ICE, the duplication and integration of the ICE at a second site in the opposite orientation and finally recombination between the two copies (Figure 3).

We used a PCR primer pair designed to amplify a fragment of the well conserved *traG *gene (Additional file 1 table S1) to screen for the presence of this type of ICE in a representative set of 31 strains of the *M. mycoides *cluster and other ruminant mycoplasmas (Table 2). Amplification products were obtained for four of the ten Mmc strains, indicating that these strains carried closely related ICE. Two additional primers (VIP-F and VIP-R), designed to flank the insertion site of one of the 95010 ICE copies, were used for PCR analysis of DNA from the same set of strains. Of the four *traG*-positive Mmc strains, three yielded the expected 1,079 bp-long amplicon, indicating that the detected ICE was integrated elsewhere on the chromosome of these strains. As expected, strain 95010 DNA did not yield any amplified product because the primers were separated by about 30 kbp and in the same orientation. The absence of amplification with "TraG" and "VIP" for the five other strains DNA was not expected; it may have been due to sequence variations that hampered the correct annealing of the primers or by other, unidentified, genetic rearrangements.

**Table 2:** Mycoplasma strains used in this study to evaluate the presence of genetic mobile elements^a^

Strain	Year	Origin	PCR				
			**IS*Mmy2***	**IS*Mmy3***	***traG***	**VIP**	**16S**
			
*M. mycoides *subsp. capri							
55507-1^b^	1998	Germany	+	-	-	-	+
95010-C1^b^	1995	France	+	+	+	-	+
Y-goat^R b^	1956	Australia	+	-	+	+	+
2002-054 (VP9L)^b^	< 2002	India	-	-	-	-	+
8756-C13^b^	< 1987	USA	-	-	-	-	+
Kombolcha^b^	1975	Ethiopia	-	-	+	+	+
WK354	1980	Switzerland	+	+	-	+	+
N108	1977	Nigeria	+	-	-	-	+
L	1975	France	+	+	+	+	+
PG3^T^	1950	Turkey	-	-	-	-	+
*M. capricolum *subsp. *capricolum*							
8086-1	1980	France	+	-	-	-	+
2002-053 (VP28L)	< 2002	India	-	-	-	-	+
California kid^T^	1955	USA	-	-	-	-	+
96038	< 1996	Greece	-	-	-	-	+
90122 (C1547)	1990	Ivory Coast	-	-	-		+
*M*. *mycoides *subsp. *mycoides *SC							
T1/44	1954	Tanzania	-	-	-	-	+
94111	1994	Rwanda	-	-	-	-	+
8740-Rita	1987	Cameroon	-	-	-	-	+
PG1^T^	< 1931	Unknown	-	-	-	-	+
*M. capricolum *subsp *capripneumoniae*							
F38^T^	1976	Kenya	-	-	-	-	+
GL102	1981	Tunisia	-	-	-	-	+
Gabes	1981	Tunisia	-	-	-	-	+
95043	1995	Niger	-	-	-	-	+
*M*. bovine group 7 of Leach							
PG50^R^		Australia	-	-	-	+	+
D424	< 1990	Germany	-	-	-	+	+
9733	1993	India	-	-	-	+	+
*M*. *putrefaciens*							
KS1^T^	1954	USA	-	-	-	-	+
Tours 2	1972	France	-	-	-	-	+
*M. yeatsii*							
GIH^T^	1981	Australia	+	-	-	-	+
*M. cottewii*							
VIS^T^	1981	Australia	+	-	-	-	+
*M. auris*							
UIA^T^	1981	Australia	-	-	-	-	+

^a ^Whenever possible, strains originating from different continents were selected to improve the representivity of the sample. PCR results are recorded as positive (+) or negative (-) with primer pairs designed to detect the presence of Insertion Sequences (ISMmy2 and ISMmy3), Integrative Conjugative Elements (*traG*) or the insertion of an integrative conjugative element at a specific location (VIP). Non specific PCR primers corresponding to 16SrDNA genes were used as positive amplification controls.

^b ^These strains were formerly identified as *M*. *mycoides *subsp. *mycoides *LC

### Mobile elements: Plasmid

Plasmid pMmc-95010 is 1,840 bp long and has a G+C content of 29.0 mole%. It is predicted to encode two proteins probably involved in plasmid replication. The protein encoded by orfA is similar to several replication (Rep) proteins of plasmids replicating by a rolling-circle mechanism. The putative pMmc-95010 Rep protein has a conserved architecture domain corresponding to that of Rep_2 plasmid replication proteins (pfam01719). The sequence included five motifs typical of pMV158 plasmid family [17]. The protein encoded by orfB has a sequence structure similar to that of the RHH_1 ribbon-helix-helix family of CopG repressor proteins (pfam1402 domain). CopG, also known as RepA, is a transcriptional regulator that controls the plasmid copy number in the pMV158 derivative pLS1 [18]. Several plasmids with sizes between 1,717 and 3,432 bp have been isolated from mycoplasmas related to the *M*. *mycoides *cluster. Plasmid pKMK1 (M81470.1) was isolated from a Mmc strain (GM12); it has a size similar to that of pMmc-95010 and the predicted proteins of the two plasmids share more than 97% similarity [19]. Complete nucleotide sequence alignment indicated nearly identical sequences. Plasmids pADB201 (NC_001382), pBG7AU (NC_002569) and pMyBK1 (NC_011102) were isolated from *M. mycoides*, *M. leachii *and *M. yeatsii*, respectively [20,21]. pADB201 and pBG7AU encode two proteins similar to OrfA and OrfB from pMmc-95010, but pMyBK1 does not. Interestingly, nucleotide alignments of the plasmid sequences suggested a composite origin of plasmid pADB201: the first thousand nucleotides are very similar to pBG7AU sequences whereas downstream sequences were closely related to sequences found in pMmc-95010 and pKMK1. This suggests that plasmids found in various mycoplasma species of the *M. mycoides *cluster may circulate among strains and recombine to generate new hybrid plasmids.

More surprisingly, blast search using pMmc-95010 nucleotide sequence identified a non-coding, GC-rich sequence of 286 nt showing 96% identity with two Mmc chromosome regions (Additional file 2 Figure S1). These regions are located within ICEs, between CDS22 and the terminal inverted repeat. Three palindromic sequences of 7, 7 and 16 nucleotides were repeated two to five times with the potential to form several stem-loop structures. A related sequence was also found in the ICE in Mmc strain GM12 and in plasmids pKMK1 and pADB201. The distribution of this trait suggests that ICEs and plasmids have probably exchanged sequences. This highly structured region covers a 130 bp sequence identified as the single-strand origin (*sso*) of pKMK1 replication (Additional file 3 Figure S2). In rolling-circle replication plasmids, the *sso *is the initiation site of lagging strand synthesis and is essential for the conversion of a single-strand intermediate into a double-strand molecule. The identification of an sso-type region in ICEs raises the question of whether this plasmid-derived sequence is involved in ICEMmc95010 dissemination.

### Mobile elements: Insertion Sequences

Five different types of insertion sequences (IS) were identified in the Mmc 95010 genome. Nine copies of IS*1296 *were identified. They were very similar to IS*1296 *copies present in the MmmSC PG1 genome both in terms of length and deduced amino-acid sequence (98% similarity). Six copies of IS*Mmy1 *were identified. The similarity with those found in MmmSC PG1 was high (87%) but lower than for IS*1296*. Six copies of a novel IS named IS*Mmy2 *were found; one of the copies was considered as the prototype for a new IS and has been submitted to GenBank (EMBL/GenBank accession N° DQ887910). This IS is 1,374 bp long, contains 24-bp inverted repeats and appears to have generated 3-bp-long direct repeats at its insertion sites. It contains a putative ribosome binding site (position 66-71) 5 bp upstream from the ATG start codon of a 1,278 bp-long orf (426 aa); the orf possesses a classical DDE catalytic site motif. Accordingly, this IS was identified as belonging to the IS*3 *family and the IS*150 *group. The presence of IS*Mmy2 *in related strains was evaluated by two techniques: Southern blotting with a DIG-labelled probe (results not shown) and PCR using a single primer corresponding to the conserved part of the inverted repeat found at the extremities of the IS element (Table 2). The results with the two techniques were entirely consistent: elements related to IS*Mmy2 *were detected in six of ten strains tested belonging to the subspecies Mmc and Mmc. However, this IS was not detected by either of the two techniques in any of four MmmSC strains. The absence of detectable IS*Mmy2 *in the MmmSC subspecies was not expected, given that this IS shares substantial similarity with two contiguous MmmSC CDS. A single DNA sequence homologous to IS*Mmy2 *was however found between positions 801579 and 802903 of the MmmSC PG1 sequence (NC_005364.2). This sequence seems to represent the remnant of an IS*Mmy2 *without transposase activity because it carries a mutation disrupting the transposase gene. The sequence found in MmmSC PG1 may thus be the result of ensuing genetic drift by accumulation of insertions, deletions (including the 49 terminal bases of the original IS) and point mutations. The failure to detect this sequence by Southern hybridization may be explained by the presence of *Hin*dIII sites in the MmmSC PG1 IS*Mmy2*-remnant sequence (the copies in Mmc IS*Mmy2 *contain no such sites) resulting in much smaller fragments. The failure to detect these sequences by PCR was clearly due to the absence of the Right Inverted Repeat essential for PCR amplification with a single primer.

Two copies of another novel insertion sequence, named IS*Mmy3 *were found. IS*Mmy3 *is 1,442 bp long with 24 nt long terminal inverted repeats that are only partially identical (66%) and has generated 3bp direct repeats. It encodes two putative polypeptides that may be fused to form a transposase as there is a potential frameshift position between nucleotide positions 503 and 510. This new IS therefore belongs to the IS*3 *family and it closely resembles IS*1296 *(60% similarity). IS*Mmy3 *was detected in three of ten Mmc strains tested but was not detected in MmmSC or Mcc strains (Table 2).

A fragment of another insertion sequence was detected (MLC_7590). This partial IS copy codes for a single protein that shares 60% similarity with transposase protein A of the IS*1296 *found in MmmSC. A very similar partial copy is also found in Mcc California kid strain (96% similarity).

**Figure 4:**
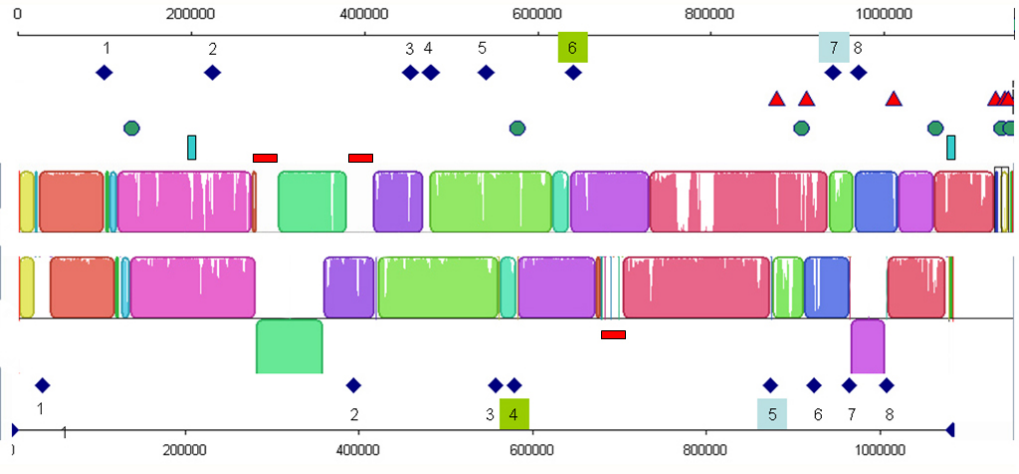
**Comparison of Mmc 95010 and Mmc GM12 genome organisation in relation to mobile elements**. The colinear blocks identified by Mauve software in the closely related Mmc 95010 and Mmc GM12 genomes are shown. The various mobile elements where then positioned on the graph. IS*1296 *copies are represented by dark blue lozenges, IS*Mmy1 *by red triangles, IS*Mmy2 *by green circles, IS*Mmy3 *by vertical blue lines and ICEs by red horizontal rectangles. Eight IS*1296 *copies are found in each genome but only two map at the same site (copies 6 and 7 in Mmc 95010). In addition, two copies of IS*1296 *are positioned on either side of an inverted DNA fragment: duplication and transposition of this IS may have been responsible for this inversion. Similarly, the duplication of the ICE in the Mmc 95010 genome may have caused the inversion of a large DNA fragment.

The diversity of IS types and copy numbers in the Mmc strains can be assessed by comparison of the GM12 and 95010 genomes (Figure 4). Both genomes contain eight copies of IS*1296 *but only two of them are inserted at the same locations. This indicates that transposition events are relatively frequent for this IS. In addition the presence of copies of IS on either side of an inverted linear block in GM12 shows that IS duplication and transposition may lead to major genome rearrangements. Note also that three other IS types (14 copies) present in 95010 do not appear to be associated with any rearrangement. The only other major rearrangement in the 95010 genome was triggered by an ICE.

### Mmc-specific clusters: the maltodextrin/maltose gene cluster

**Figure 5:**
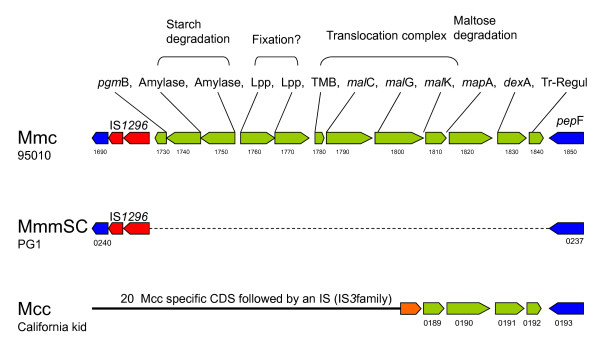
**Organisation of the "maltodextrin/maltose gene cluster" in the Mmc 95010, MmmSC PG1 and Mcc California kid genomes**. In the Mmc 95010 genome, the "maltodextrin/maltose gene cluster" is composed of 12 CDS shown in green. The cluster is flanked by an IS*1296 *copy and *pep*F. The cluster comprises: a phosphoglucomutase (*pgm*B), two amylases (CDS 1740 and 1750), two lipoproteins that may be involved in binding (CDS 1760 and 1770), a translocation complex (CDS 1780, *mal*C, *mal*G, *mal*K), two CDS involved in maltose degradation (*map*A, *dex*A), and a CDS probably involved in the regulation of transcription. This cluster is absent in the PG1 genome although the flanking genes (the IS*1296 *copy and *pep*F) are still present. In the Mcc California kid genome, a large portion of the cluster has been deleted, (8 CDS out of 12) and been replaced by 20 CDS followed by an IS*3 *copy.

**Figure 6:**
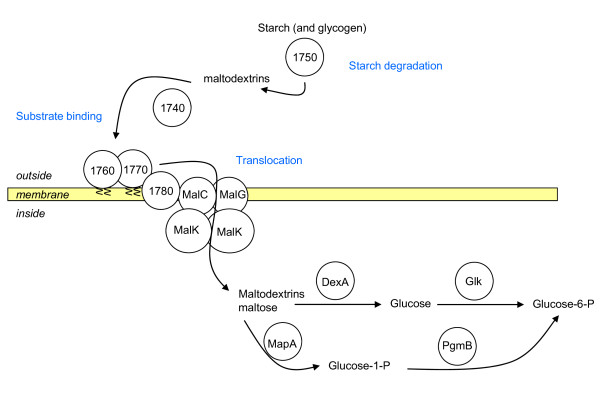
**Putative model for starch, maltodextrin and maltose utilization in *M. mycoides *subsp. *capri***. The proteins involved are shown as circles with their relevant gene names or CDS numbers. Starch is hydrolyzed by amylases extracellularly to give maltodextrin and maltose. The resulting oligosaccharides are transported into the cytoplasm by a translocation complex. Further degradation of the sugar leads to glucose-6P which can enter glycolysis (adapted from [22]).

A specific stretch of 12 CDS was identified downstream from an IS*1296 *copy (MLC_1730 to 1840). None of these CDS have any significant similarity with the PG1 genome. All have similarities with genes involved in carbohydrate metabolism and more specifically starch/glycogen and maltose utilization (hereafter named the maltodextrin/maltose gene cluster) (Figure 5). This cluster is composed of genes coding for a phosphoglucomutase (EC5.4.2.6), amylases that may be involved in starch degradation, two lipoproteins that may be involved in substrate binding, a hypothetical protein with transmembrane segments, followed by *malC, malG*, and *malK *genes that may encode a translocation complex, *mapA *(EC2.4.1.8) a maltose phosphorylase, *dexA *(EC3.2.1.10) a gene encoding oligo-1,6 glycosidase and, at the end, a transcription regulator. Most of these genes are similar to genes found in other bacteria such as *Listeria*, *Mycoplasma *(*M. mobile *and *M. pulmonis*), and *Lactococcus*, but the most similar homologues were mostly in *Bacillus *in which maltose and maltodextrin utilization has been documented [22]. A tentative model for starch and maltodextrin utilization can be established for Mmc 95010 with the two lipoproteins encoded by the cluster possibly involved in substrate binding (Figure 6) and MLC_1780 in transmembrane transport. Only four genes of this operon were found in the MccCalifornia kid genome and none were found in MmmSC PG1 although the flanking genes were present (IS*1296 *and *pep*F). This suggests that this cluster was present in the ancestor of the *M*. *mycoides *cluster and that the whole cluster has been deleted from MmmSC PG1. For Mcc California kid, part of the cluster has been deleted and replaced by 20 CDS found inserted at this position. The maltodextrin/maltose gene cluster is fully conserved in the Mmc GM12 genome but there is no IS*1296 *copy found upstream.

### Lipoproteins

The number of predicted lipoproteins (Lpp) was strikingly higher for Mmc 95010 (N = 86) than for PG1 (N = 56). Sixty of these Lpp in Mmc 95010 can be grouped into 16 families, and seven are organised in clusters (Additional files 4, 5 and 6, figures S3, S4 and S5). Many of these clustered Lpp are absent from MmmSC PG1 or are interrupted by IS elements. In contrast, all these clusters were found in strain GM12 with a perfect synteny with the 95010 genome. However, the similarity (between GM12 and 95010) differed greatly from one Lpp to another (from 52 to 98%), whereas the flanking housekeeping genes were well conserved (mean of 98% similarity). For example, the five Lpp illustrated in Additional file 5 Figure S4 (MLC_9030 9040; 9050; 9070; 9080; 9090) form a family with a highly conserved signal peptide and lipoprotein cleavage site (AVIAC) and some conserved stretches in their C terminal parts. The presence of IS at this locus may be an indication that these elements played a role in the duplication of these genes. Similar Lpp are present at the same location in the GM12 genome (MMCAP2_0900 to 0904) and were identified as putative variable lipoproteins. The similarity of these 95010 Lpp with the GM12 Lpp was between 65 and 93%. These Lpp also shared similarities with a cluster of Lpp found in Mcc (MCAP_0593; 0594; 0595 and 0596), identified as "Vmc". They vary in length and display repeated AA motifs which are typical of variable lipoproteins in mycoplasmas. Such repeated motifs were not identified in the corresponding 95010 and GM12 Lpp. Differences in lipoprotein expression may be linked to the length variation of poly TA tracts within the promoter region of the Lpp.

This phase variation has been described for Vmm lipoproteins in MmmSC strains [23]. Poly-TA tracts with more than ten repeats were found at five positions in the PG1 chromosome. Three of these positions are immediately upstream from genes encoding Lpp (MSC_0117; 0390 and 1005) and the two others are near to transposase genes. Such poly-TA tracts are also found in the 95010 genome, at six positions. All precede Lpp genes and the number of TA repeats differs: MLC_1150 (N = 10); MLC_5200 (N = 10); MLC_9030 (N = 12); MLC_9070 (N = 16); MLC_9120 (N = 15); MLC_9140 (N = 15). A LC-MS/MS proteomic approach allowed the detection of 30 lipoproteins in 95010 and only 18 in MmmSC PG1, and thus a similar proportion (about one third) of the predicted genes in each strain. Of the six Lpp at the locus shown in Additional file 5 Figure S4, only two were detected.

One Mmc 95010 Lpp family, with genes downstream from an IS*1296 *element and that shares some similarity with the spmA family in *M. agalactiae*, is not present in MmmSC [15,16] (Additional file 6 Figure S5). In *M. agalactiae*, the genes of the homologous family are also clustered and most of them are preceded by G-rich stretch that may be involved in the regulation of expression. Interestingly, in the Mmc 95010 genome, intergenic regions upstream from these genes contain a conserved GC(T)_17-20 _motif 88 to 113 nt upstream from the ATG start codon. A similar motif (GC(T)_16_) is present 97 nt upstream from the ATG of the unique member of the homologous family in Mcc. The function of these motifs that are unique in these highly AT-rich genomes, like the function of *M. agalactiae *G-rich repeats, is unknown.

Three genes involved in lipoprotein processing were identified in Mmc 95010: two encoding diacylglyceryl transferases (*lgt*: MLC_8480 and MLC_8500) and one a lipoprotein signal peptidase (*lsp*: MLC_5520). By contrast, only one *lgt *was found in the MmmSC PG1 genome; the gene orthologous to the second *lgt *is interrupted by a frameshift mutation resulting in two "hypothetical proteins" (MSC_0936 and 0937) that are not identified as *lgt *or pseudogenes in the current annotation of the MmmSC PG1 genome. Phylogenetic analysis of diacylglyceryl transferase across mollicutes reveals that most species possess only one *lgt *gene but that duplication has occurred in the ancestor of the Spiroplasma phylogenetic group. Whether the inactivation of one copy in MmmSC PG1 has affected the lipoprotein processing in this strain is not known.

### Pseudogenisation in MmmSC PG1 and genes interrupted by mobile elements

The annotation process of the Mmc 95010 genome identified 45 CDS that were highly similar to two or three contiguous CDS in MmmSC PG1. These MmmSC PG1 CDS correspond to pseudogenes (N = 96) that were not identified as such at the time of the annotation because no sequence from a related mycoplasma was available (Table 3). The proteomic approach allowed the detection of peptides for 11 of these 45 Mmc 95010 genes. Surprisingly, five products from the corresponding interrupted MmmSC genes were also detected. These products may result from the expression of a partial gene. Alternatively, there may be errors in the MmmSC PG1 sequence. In addition to disruption of genes by frameshift mutations, five PG1 genes were truncated by insertion sequences. Only three of these five genes had orthologues in Mmc (two CHP and one putative haemolysin). Conversely, four CDS in Mmc 95010 are interrupted by IS elements but only one of them was similar to a gene in MmmSC PG1 (a lipoprotein). Three Mmc 95010 genes (MLC_1870, 3270, 4830, coding for a leucylpeptidase, a protease and an endopeptidase, respectively) had orthologues in MmmSC PG1 that are altered: the MmmSC leucylpeptidase gene is truncated, giving two CDS (MSC_0234, MSC_0235) by a frameshift mutation; the protease gene is interrupted by an insertion sequence (MSC_0343); and the endopeptidase (MSC_0504) gene is shorter by 100 codons than the Mmc 95010 ortholog. The inactivation of these three genes may explain why MmmSC PG1 is less able to hydrolyse proteins as evidenced by the inspissated serum digestion test (Table 1).

**Table 3:** Frameshift mutations in MmmSC PG1

Mmc CDS N°	Detection of Protein ^a^	Product (Mmc)	MmmSC CDS	Detection of Protein ^a^
0210	-	recG	0022, 0023 and 0024	-
0350	-	CHP	0036 and 0037	-
0370	-	CHP	0040 and 0041	-
0630	-	TMB protein	0075 and 0076	-
1000	+	lipoprotein	0103 and 0104	+
1500	-	lipoprotein	0169, 0170 and 0171	-
1910	-	TMB protein	0227 and 0228	-
1870	+	pepA	0234 and 0235	-
1650	-	CHP	0242 and 0243	-
2550	-	lipoprotein	0285 and 0286	-
2540	-	lipoprotein	0285 and 0286	-
2530	+	TMB protein	0287 and 0288	+
2330	-	protein phosphatase	0307 and 0308	-
2320	-	TMB protein	0309 and 0310	-
2290	-	TMB interrupted by ICE	0314 and 0315	-
3100	-	lipoprotein	0317 and 0318	-
3270	-	TMB and protease	0343 and 0344	-
3280	-	lipoprotein	0345 and 0346	-
3560	-	CHP	0378 and 0379	-
3790	-	TMB protein	0399 and 0400	-
4410	-	putative abc transporteur	0459 and 0460	-
4510	-	TMB protein	0470 and 0471	+
4520	+	TMB and efflux	0472 and 0473	+
4840	-	endopetidase O	0504	-
4930	-	TMB substrate transport	0513, 0514 and 0515	-
4980	-	TMB protein	0521, 0522 and 0523	-
5170	-	TMB protein	0536 and 0537	-
5250	+	CHP	0545 and 0546	-
5570	-	CHP	0589 and 0590	-
5940	-	lipoprotein	0631 and 0632	-
5970	-	TMB protein	0636 and 0637	-
6080	+	CHP	0652 and 0654	-
6100	+	CHP	0656, 0657 and 0658	-
6510	-	TMB protein	0694 and 0695	-
6580	-	TMB protein	0705 and 0706	-
6620	+	lipoprotein	0710 and 0711	+
7000	-	transcriptional regulator	0750 and 0751	-
7010	-	TMB protein	0753 and 0754	-
7200	+	lipoprotein	0772 and 0773	-
7340	+	HAD hydrolase	0792 and 0793	-
7420	-	CHP	0819 and 0820	-
8740	-	TMB protein	0919 and 0920	-
8500	-	lgt	0936 and 0937	-
8860	+	CHP	1012, 1013 and 1014	-
8970	-	TMB protein	1023, 1024 and 1025	-

a "+" indicates that the protein was detected by LC-MS/MS with at least 2 disctinct peptides. MmmSC CDS should be considered as pseudogenes unless there was a sequencing error leading to an artefactual frameshift mutation.

**List of CDS with frameshift mutations in the MmmSC PG1 genome as evidenced by comparison with Mmc CDS**.

The insertion of these 2 ICE copies in the Mmc 95010 genome has disrupted two CDS. The first encodes a putative lipoprotein (MLC_2070; 142 AA) whose orthologue in Mmc GM12 (MMCAP2_0208) is 263 AA long. The second encodes a putative permease, 1624 AA long, annotated as a pseudogene in 95010 (MLC_2290), whose orthologue in GM12 (MMCAP2_0272) is 1773 AA long and possesses two FtsX domains that may be associated with lipid transport. A gene orthologous to this permease was also found in the MmmSC PG1 sequence (MSC_0033, 1796 AA long).

### Global comparison of gene repertoires

**Figure 7:**
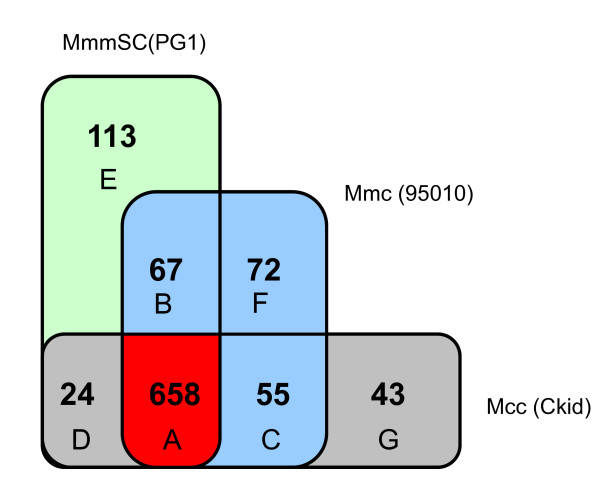
**Number of orthologous genes in the Mmc 95010, MmmSC PG1 and Mcc California kid genomes**. Numbers of orthologous genes in Mmc 95010, MmmSC PG1 and Mcc California kid genomes were evaluated using the MBGD database http://mbgd.genome.ad.jp/. The core genome of these three strains consists of 658 genes. Letters refer to supplementary table S2 giving the list of orthologues.

A search for orthologous genes in the Mmc 95010, MmmSC PG1 and Mcc California kid genomes identified various clusters of orthologs that were specific to each genome or shared by two or three genomes (Figure 7 Additional file 7 table S2).

The majority of the 113 PG1-specific clusters (Additional file 7 table S2, sheet E), correspond to hypothetical proteins and predicted lipoproteins, most (63%) of which, however, are considered to be pseudogenes. In addition, two clusters identified as an Aspartate-tRNA ligase (*asp*S, MSC_0432) and a CTP synthetase (pyrG, MSC_0902) are in fact partial sequences that were erroneously annotated and should also be considered as pseudogenes. MSC_0902 maps near two other CDS that are duplicated, probably when an IS element transposed (IS*1296 *copy). The same event may have occurred for three clusters identified as *cps*, *glf *and *gal*E (MSC_0970; 0974 and 0985) which are partial duplicated copies of genes with orthologues in 95010. These sequences should also have been annotated as "putative pseudogenes". In addition, PG1-specific clusters included restriction modification genes, notably an adenine or cytosine methylase (MSC_0950 and 0951).

As expected, some transposase genes and genes belonging to the maltodextrin/maltose gene cluster were identified as Mmc 95010-specific CDS (Additional file 7 Table S2, sheet F). One CDS coding for a predicted MatE efflux family protein (Multi Antimicrobial Extrusion) was identified (MLC_6090). These proteins mediate resistance to a wide range of cationic dyes, fluroquinolones, aminoglycosides and other structurally diverse antibodies and drugs, and most possess 12 alpha-helical transmembrane regions. This is the case for MLC_6090 in strain 95010 but apparently not in strain GM12 in which the CDS has been disrupted resulting in two CDS which should be considered to be pseudogenes.

Some ribosomal proteins, such as *rpm*J, *rps*S, *rps*N and a preprotein translocase *sec*G, were identified as specific for a subset of strains (Additional file 7 Table S2, sheet C). This may be a consequence of the very small size of these proteins which resulted in very low blast e values or, more frequently, of incorrect annotation of the PG1 genome. This could be evidenced by a BLASTN search that identified *rpm*J and *rps*N in the PG1 sequence at positions 830855 and 836897, respectively, and the identification of *sec*G at position 973088. The 658 CDS clusters that were found as orthologous in all of MmmSC PG1, Mmc 95010 and Mcc California kid (Additional file 7 Table S2, sheet A) may be considered as the "*M*. *mycoides *core genome".

A blast analysis of whole CDS in Mmc strains 95010 and GM12 showed that 80% of these CDS shared more than 90% identity. This illustrates the very close relatedness of the two genomes and confirms their classification as belonging to the same subspecies. The divergent CDS had identity values that varied from 20 to 90% (Additional file 8 Table S3). Many corresponded to partial sequences that may be considered as pseudogenes and the lower identity values may be due to these sequences being short or to the fact that mutations are likely to accumulate more rapidly in pseudogenes than in functional genes. Other divergent genes were identified as lipoproteins and the percentage of identity for these genes varied from 30% to 90%. Similar polymorphism was also evidenced in membrane surface expressed proteins highlighting the highly dynamic and diverse surface architecture of otherwise closely related strains. This is undoubtedly a consequence of environmental pressures that shape the variability of these surface exposed proteins. This variability also explains why reference identification tests such as the growth inhibition test may give such variable results as hyperimmune sera contain large amounts of antibodies directed towards these immunodominant antigens. Divergent CDS also included restriction and modification genes such as "C5 Cytosine DNA methylase" (MMCAP2_0554), "GCATC recognizing Type II restriction modification system" (MMCAP2_2020; 0900 and 0920), "restriction endonuclease" (MLC__1620), "modification methylase" (MLC_1630), "cytosine-specific DNA methyltransferase *Sau*96I" (MLC_2020) and "Type II site specific deoxyribonuclease (*Sau*96I like)" (MLC_2030).

## Discussion

Our goal was to compare the genomes of two very closely related Mycoplasma subspecies and accurately determine their degree of relatedness. This can be done at various levels by comparing the genome organization, the gene repertoire and the polymorphisms within the genes.

Genome plasticity in mycoplasmas of the mycoides cluster is greatly influenced by the "mobilome" and more specifically by ICE and IS. Such elements were evidenced in the 95010 genome and have been shown to drive overall genome plasticity.

Integrative conjugative elements have a modular structure and contain blocks of genes dedicated to integration into and excision from the chromosome, as well as conjugal transfer [24]. Until recently, ICEs had been evidenced in a limited range of hosts belonging to the four major divisions of bacteria. However, whole genome sequencing projects suggest that ICEs are widespread in bacteria and could be one of the main types of shuttle for horizontal gene transfer [25]. ICEs have now been identified in various *Mycoplasma *species, including *M. fermentans*, *M. agalactiae *and Mcc [16,26,27]. The ICE copy number in mycoplasmas seems to be small, for example only four copies of two ICE types in *M. fermentans*. ICE copy numbers are much higher in other bacterial species: in *Orienta tsutsugamushi *duplicated elements, including ICEs, account for more than 37% of the genome [28,29]. Our analysis of the Mmc genome shows that these elements have a direct impact on genome rearrangements, although the exact mechanisms leading to excision, integration and/or conjugation to another cell remain to be elucidated. In the genus *Bacillus*, transfer of ICE copies seems to be favoured by high densities of cells not carrying these elements and integration into a cell apparently leads to blocking the entry of additional copies [30,31]. The possibility that these elements are involved in transferring virulence factors to and between mycoplasmas needs to be investigated.

Comparison of plasmids from the mycoides cluster suggested that various recombination events may have occurred during the spread of these plasmids among strains. More surprisingly, alignments of plasmid and ICE sequences in Mmc 95010 indicated that these mobile elements may have exchanged sequences. This new finding suggests that these two types of mobile elements could interact within mycoplasma cells and maybe even cooperate in transmission from cell to cell.

Insertion sequences are another driving force for genome plasticity in the mycoplasma mycoides cluster. Comparisons of genomes revealed substantial diversity of IS type and copy number even between closely related strains (95010 and GM12) and that duplication of IS copies may lead to large DNA fragment inversions. This contrasts with the findings for two *M. agalactiae *genomes in which the presence of 15 IS copies and three ICE copies was not associated with any large-scale genetic rearrangement [15]. In the case of the *mycoides *cluster, the major contribution of IS to genome plasticity is well illustrated by the comparison between Mmc and MmmSC genomes. The MmmSC PG1 genome has large numbers of IS copies, IS*1634 *having the highest copy number (N = 60). These IS-elements have not only led to large DNA fragment inversions but also large DNA fragment duplications and deletions. This is not unprecedented in the bacterial world and IS expansions may result from an evolutionary bottleneck due to bacterial population isolation [32]. In the case of MmmSC PG1, this bottleneck may have been associated with the strict adaptation of this subspecies to the bovine lung. Indeed, IS*1634 *shares 97% identity with IS*Mbov3 *which is found in *M*. *bovis*, a very common pathogen isolated from cow lungs [33]. This close relatedness certainly indicates recent HGT and the absence of IS*1634 *from Mmc suggests that this IS was acquired by MmmSC from *M. bovis*. Such exchanges of IS in between the *mycoides *cluster and the *M. bovis/M. agalactiae *cluster have already been proposed [33].

A striking feature of the MmmSC PG1 genome, as compared to those of Mmc 95010 and GM12, is the large number of pseudogenes in the vicinity of IS elements. Altogether, more than 98 MmmSC of the originally described putative genes are certainly pseudogenes as a result of frameshift mutations or inserted insertion sequences. This represents more than 9% of the total number of MmmSC genes that were annotated in 2004. High percentages of pseudogenes are often associated with a recent adaptation to a host and to virulence, as suggested for *Yersinia pestis *[34]. Adaptation to a new host allows a massive clonal population growth in which all mutations affecting genes that are not essential for bacterial survival in the new environment are maintained. Such clonal expansion also explains the limited polymorphism of the housekeeping genes. Reductive evolution of this type has been described for various pathogens in addition to *Yersinia pesti*s, including *Orienta tsutsugamushi*, the agent of scrub typhus, *Ricketsia prowazeckii*, the agent of epidemic typhus and *Aliivibrio salmonicida*, the agent of cold-water vibriosis [29,35,36]. In the case of MmmSC, adaptation to a new host may also have favoured the acquisition and multiplication of new IS types, such as IS*1634*. Similarly, pseudogenisation was also observed in *M. bovis*, where an adhesin of *M. agalactiae *was inactivated upon infection of a different host [37]. Longer evolution times would possibly allow a streamlining of the genome with a reduction of the number of pseudogenes by a deletion process. Mmc is an ubiquitous pathogen that is present in numerous species (sheep, goats) all around the world; it is an opportunist pathogen that can infect diverse organs and can even be found in the ear canal of healthy goats (or in parasites found in the ear canal). By contrast, MmmSC is strictly pathogenic and limited to a single host, cows, and to a single organ, the lung. This is consistent with an Mmc ancestor, adapted to various ecological niches in small ruminants, adapting to a bovine host where it colonizes only the lungs, and evolving into what is now known as MmmSC. Genomic studies, and particularly the observation that intraspecies polymorphism in housekeeping genes is much more limited in MmmSC than in Mmc, support the hypothesis that MmmSC emerged only recently [38].

The availability of whole genome sequences may help unravel the genetic events underlying phenotypic diversity among closely related strains. As an example, the utilization of maltose in the *M. mycoides *cluster species has been studied by AbuGroun [39]: no maltose utilization was found in MmmSC. Maltose is utilized by Mcc and more rapidly by Mmc. However, some Mmc strains failed to metabolize maltose at all. The presence of an alpha glucosidase was also detected by a rapid colorimetric test using pNPG flooded on mycoplasma colonies [40]. None of the MmmSC strains tested possessed any glucosidase activity although most Mcc and Mmc strains did. However MmmSC strains express beta-glucosidases with variations which may be related to cytotoxicity [41]. Our findings are in accordance with these observations. What remains to be verified is the integrity of the maltose gene cluster in the Mmc strains that fail to utilize this substrate, and the ability of Mmc strains to utilize starch. Mcc California kid is expected to be unable to use starch, although other Mcc strains should be able metabolize this carbon source.

Surface proteins and more specifically lipoproteins that play key roles in interactions with the environment are determinant for the lifestyle of mycoplasmas. They contribute to the uptake of nutrients and can mediate essential functions during the infection cycle. Some play a role in cytadhesion, and other bind IgAs to allow the cells to escape cellular recognition [42]. Surface proteins can also display mechanisms of phase variation as a means to escape the host immune responses [43]. At the same time they are excellent immunogens, their lipid moiety acting with adjuvant-like proinflammatory activity and their protein part evoking an immune response [44]. However, the type of immune response they trigger may vary according to the Lpp involved. In the case of MmmSC PG1, LppA seems to trigger a cellular response, involving CD4 cells producing interferon gamma, whereas LppQ, LppB and LppC do not [45]. The presence of 86 genes coding for Lpp in the Mmc 95010 genome, as compared to 56 in MmmSC PG1, is in agreement with findings in other mycoplasma species. The number of Lpp genes in two strains of *M. agalactiae *is 100 and 67, the latter number being that of reference strain PG2 [15]. In *M. agalactiae*, poly-G tracts are suspected to be involved in genomic rearrangements and possibly in the control of expression of genes in the region encoding the so-called spma lipoprotein family [15,16]. A locus encoding homologous predicted lipoproteins was found in Mmc 95010 with intergenic regions containing GC(T)_7-20 _motifs. This suggests that there may have been exchange of genes belonging to this family between these ruminant pathogens followed by divergent evolution of intergenic motifs and subsequent expansion of the gene families. In accordance with this hypothesis, only one single member of this family was found in Mcc genome whereas variable expansions were observed in the two strains of *M. agalactiae*. In *M. mycoides*, intergenic nucleotide tracts are found at other loci. Poly-TA tracts, with more than 10 repeats, were found at six locations both in the 95010 and the GM12 genomes. The size of these tracts differed between strains. However, these size variations should be interpreted with great care as most sequencing projects use cloned bacterial stocks: such variants may differ from the main population. In 95010, the percentage of Lpp that were detected by the proteomic analysis was 30%. This is slightly lower than reported by other studies in which amphiphilic proteins were first concentrated by Triton X-114 extraction [15]. As a consequence, the differences may simply be due to differences in the sensitivity detection of the techniques used. In addition, Lpp expression may be driven by environmental conditions and our results apply only to mycoplasmas grown in rich media, *in vitro*. Co-incubation and adhesion to cells may well trigger the expression of a different set of Lpp as has been demonstrated for *M. pneumoniae *in contact with lung epithelial cells [46]. This type of modulation of expression may play an important role in virulence.

In fact, the evolution of MmmSC genome may be shaped by unconstrained population growth in infected animals, followed by extreme transmission bottlenecks from host to host. Furthermore, current MmmSC strain populations may also be shaped by CBPP control strategies based on slaughter and vaccination. Existing MmmSC strains may well have adapted to this artificial selection that has been implemented for more than 100 years. The MmmSC genome is larger than that of Mmc, mostly due to gene duplications, and the insertion of multiple copies of Insertion Sequences. IS elements seem to play a prominent role in this gene rearrangement process, as demonstrated during growth *in vitro *under conditions of stress induced by high temperature (41.5°C) [47]. Fever in CBPP-infected animals may induce a similar stress and favour gene rearrangements in MmmSC. However, the MmmSC genome is also characterized by a high degree of gene decay with more than 9% of the originally described genes likely to be pseudogenes. Many of these genes are not associated with any known function ("ORFans"), consistent with the notion that the number of genes in whole genomes is often overestimated [48]. This fits also well with a non-adaptative genomic complexity theory allowing duplications or pseudogenes to be maintained in the absence of an adaptive selection that would lead to purifying selection and genome streamlining [49,50].

Genome structure in both of the subspecies seems to have been affected by mobile genetic elements despite these elements differing in kind and in numbers. Integrative conjugative elements have been identified in Mmc where they were shown to induce chromosomal rearrangements, but not in MmmSC. They may also have played a role in gene acquisition although this has not yet been demonstrated. Insertion sequences were identified in both subspecies but, here again, there are differences: Mmc and MmmSC have only two IS types in common and MmmSC possess only three IS types present in large copy numbers (95 copies) whereas Mmc possess five IS types but only in lower copy numbers (24 copies). Again the larger copy number in MmmSC may be associated with an evolutionary bottleneck such that they provide transitory selective advantages to their host such as HGT and genomic rearrangements [32].

Homologous recombination has been demonstrated experimentally in Mcc and Mmc strains [51]. This does not seem to be the case in MmmSC where multiple attempts to obtain homologous recombinations *in vitro *have failed [52]. These failures could be linked to the functional absence of two genes, *recG *and *recR*, which are disrupted by frameshift mutations.

## Conclusions

Comparing the genomes of two subspecies allows a micro-evolutionary analysis. Genome evolution is expected to be directly linked to the ecological niche of the two organisms and the driving forces shaping chromosome organization, gene content and sequence evolution. Preliminary data, using multilocus sequence typing, had already shown that Mmc strains were much more diverse than MmmSC strains [38]. This is consistent with the different ecology of the two organisms and the associated population dynamics.

The comparison of the Mmc and MmmSC genomes has revealed their very close relatedness, especially evident from the sequence similarities in their housekeeping genes. However, in view of the earlier belief that the two subspecies were nearly indistinguishable, there is a surprisingly large number of differences. Many of the differences are associated with genes of unknown function, and many of these genes may have been acquired by HGT. Note also that comparison of single strain genomes is limiting because a single genome cannot reflect the gene repertoire of a species. In addition, the comparison with MmmSC PG1 could lead to some bias as this strain is a "laboratory" strain which has lost its pathogenicity and its genome has been subject to genetic drift since its, unknown, time of isolation. Understanding the real driving forces of genetic fluidity will certainly need further sequencing, whole genome assemblies and re-annotation of many well-characterised field strains, not only of Mmc and MmmSC but also of closely related species or and other bacterial species that share the same ecological niche.

## Methods

### Bacterial strains

Mmc strain 95010 was isolated from a young female goat (alpine breed) with polyarthritis in February 1995 near Bourges (France). It was identified, at the time, by biochemical tests (Digitonine+, film and spots -, glucose+, tetrazolium reduction +, arginine - and phosphatase +) and by growth inhibition tests (positive with antiserum against strain YG and negative or partial with antisera against strains PG3, PG1, PG50, F38, California kid). It was subsequently characterized by multilocus sequence typing with *fus*A, *glp*Q, *gyr*B, *lep*A and *rpo*B partial gene sequences [38]. The strain was cloned to ensure its purity and "clone C1" was used thereafter. Other strains were included in the study (see Table 2 for the full list), notably to investigate the presence of repeated elements in various strains of each species or subspecies of the "mycoides cluster" or species that are found in ruminants.

### Sequencing and annotation

The complete sequence of Mmc 95010 was obtained by a shotgun strategy. To prevent cloning bias, three plasmid libraries were obtained after mechanical shearing of genomic DNA and ligation of 3 kb (A) and 4 kb (D)/10 kb (B) fragments into pNAV (derived from pcdna2.1) and pCNS (derived from pSU18) vectors, respectively. In addition, large inserts (25 kb (C)), generated by *Hin*dIII partial digest, were introduced into pBeloBAC11. Vector DNAs were purified and end-sequenced (n = 15744 (A), n = 11424 (B), n = 1536 (C), n = 5184 (D)) using dye-terminator chemistry on ABI3730 sequencers. To reduce repeated sequence assembly problems, a pre-assembly was performed using the Phred/Phrap/Consed software package http://www.phrap.com[53]. The finishing step was achieved by primer walks and PCR and transposon bomb libraries. A total of 990 sequences were needed for gap closure and quality assessment.

Genome annotation was performed using the CAAT-Box platform which was customized to facilitate the annotation process [54]. CDS were first detected using the Genemark software implemented in the CAAT-Box environment [55]. Putative CDS of more than 300 amino acids were used to train the Markov model (order 5). The three codons AUG, UUG, and GUG were used as potential start codons, and UAG and UAA were defined as stop codons. Once trained, the Markov model was applied to the complete genome using 80 bp as a cut-off value for the smallest CDS. Prediction of CDS with CAATBox also integrates results of BLAST searches to discriminate highly probable CDS from false ORFs [56]. The databases used for this purpose were SwissProt http://www.ebi.ac.uk/swissprot/index. html, trembl http://www.ebi.ac.uk/embl/index.html, and MolliGen http://molligen.org, a database dedicated to the comparative genomics of mollicutes [57]. To determine the extent of sequence similarity, alignments between predicted proteins and best BLAST-hit sequences were performed using NEEDLE software implementing the Needleman-Wunsch global alignment algorithm and using the BLOSUM62 matrix [58]. During the annotation process, proteins were considered to be homologues if the similarity in these alignments exceeded 40%. Predicted proteins with lower or only local similarities with previously characterized proteins were annotated as hypothetical proteins. Most start codons were identified according to CAAT-Box recommendations that resulted from both Genemark coding state prediction and BLAST results analysis. For CDS showing neither obvious homology relationships nor clear coding curves, the most upstream start was chosen, with a preference for the most frequently used AUG codon. Other tools incorporated into CAAT-Box were also used to improve annotation and function predictions: they included Inter-ProScan [59] and PrositeScan for domain detection and TMHMM for trans-membrane segments prediction [60,61]. To recover small CDS or gene fragments that may have been discarded during the CDS prediction process, intergenic sequences of more than 80 bp were systematically compared to reference databases using BLASTX. The annotation of each CDS was manually verified by at least two annotators. The tRNAs were mapped on the chromosome using tRNAscan-SE software and the rRNA genes were identified by BLASTN searches for homology with rRNA genes in MmmSC [62]. Precise boundaries were established after comparisons with the sequences stored in the European Ribosomal RNA Database http://www.psb.ugent.be/rRNA and the 5 S Ribosomal RNA Database http://www.man.poznan.pl/5SData[63,64]. The *rnp*B gene of the RNase P system and the tmRNA were sought by BLASTN searches for sequence similarity with homologues from Mcc and MmmSC, respectively.

### Whole genome structure comparison

Mmc 95010 genome was aligned with those of MmmSC PG1, Mmc GM12 and Mcc California kid with Multiple Genome Alignment software MAUVE v2.2.0 http://gel.ahabs.wisc.edu/mauve[65]. This software allows the identification of locally collinear blocks (LCB) that are conserved segments that appear to be internally free from genome rearrangements. Genomes were displayed on a map along lines representing the whole sequences with LCBs of various colours. Each of these block outlines surrounds a region of the genome sequence that aligned to part of another genome. When a block lay above the centre line, the aligned region was in the forward orientation relative to the first genome sequence. Blocks below the centre line indicate regions that aligned in the reverse complement (inverse) orientation. Regions outside blocks lacked detectable homology among the input genomes.

### Mobile elements

Integrative conjugative elements (ICE) were identified by homology search and by similarity with other ICEs previously identified in mycoplasma species.

Insertion Sequences (IS) in the Mmc genome were identified during annotation. The total number of IS copies was identified by "auto-blast" in the CAAT-box platform and by using 40 bp-long motifs taken at the extremities and in the middle of each IS type. Search for these motifs with Vector NTI (v:10.3.1) (Invitrogen) allowed the identification of additional truncated copies that may have been missed during annotation. The positions of repeat elements in the PG1 sequence were also retrieved from the MolliGen database. The IS copies were then positioned on a ruler along the genome and placed on the MAUVE alignment. New IS types have been submitted to the dedicated web server "IS Finder" http://www-is.biotoul.fr/.

### Gene repertoire, homologies and orthologous search

Orthologous genes in Mmc 95010, MmmSC PG1 and Mcc California kid were investigated using the MBGD database http://mbgd.genome.ad.jp/ with standard parameters [66,67]. A BLASTP comparison was performed with all CDS from Mcc 95010 and Mcc GM12 to evaluate the similarities at each CDS level. The distribution of similarity results allowed the identification of CDS that were specific for each of the two genomes.

### Proteomics

Strains MmmSC 8740-Rita and Mmc 95010 were grown in Hayflick medium at 37°C and harvested by centrifugation at the late exponential phase of growth. Whole mycoplasma pellets were washed twice in PBS and then solubilized in Laemmli buffer. Samples were subjected to electrophoresis in a 10% SDS acrylamide gel. Gels were sliced, treated with acetonytril, dried, and digested with trypsin. The resulting peptide mixtures were concentrated and analyzed on a Dionex U-3000 Ultimate nano LC system coupled to a nanospray LTQ XL IT mass spectrometer (Thermo-Finnigan, San Jose, CA). Data were acquired in a data dependent mode alternating an MS scan survey over the range m/z 300-1700 and three MS/MS scans in an exclusion dynamic mode. Data were searched by SEQUEST through Bioworks 3.3.1 interface (Thermo-Finnigan) against the CDS of MmmSC PG1 and the CDS of Mmc 95010. DTA files were generated for MS/MS spectra that both reached a minimal intensity, 1.10e3, and a sufficient number of ions, 10.

## Authors' contributions

FT and AB designed the initial genome sequencing project. VB and BV carried out the DNA libraries, sequencing, finishing and assembly of the 95010 sequence. FT, LMS and PSP carried out the genetic analysis and manual expert annotation of the genome. WS and VD analyzed the distribution of Insertion Sequences and ICE copies. AML carried out the LC-MS/MS whole proteomic sequencing. MB analyzed the plasmid and ICE sequences. DJ adapted the bioinformatic server used for the annotation. FT and PSP drafted the manuscript. FT, PSP and AB revised it. All authors read and approved the final manuscript

## Supplementary Material

Additional file 1 **"PCR primers used during this study"**. This additional table lists the names and sequences of primers used in the study. Click here for file

Additional file 2 **"Comparison of related plasmid and ICE sequences in Mmc 95010"**. This figure describes the positions and sequences shared by the integrative conjugative elements copies and the plasmid that was characterized in the same strain. Click here for file

Additional file 3 **"Comparison of plasmids from the mycoides cluster"**. This figure depicts the results of a three-way BLASTN comparison of three mycoplasma plasmid sequences using the Artemis Comparison Tool [71] Click here for file

Additional file 4 **"Organization of lipoprotein gene clusters in the Mmc 95010 genome"**. This figure is a schematic representation of 6 lipoprotein families (A to F) found in the genome of strain 95010. Click here for file

Additional file 5 **"organization of a Mmc-specific lipoprotein gene clusters in the Mmc 95010 genome"**. This figure is a schematic representation of a lipoprotein family found in strain 95010 genome but not in the published MmmSC genomes. Click here for file

Additional file 6 **"Peculiar Mmc genomic locus encoding predicted surface proteins"**. This figure is a schematic representation of the locus and homologies with *M. agalactiae *or *M. capricolum *subsp. *capricolum *genes. Click here for file

Additional file 7 **"List of gene clusters identified as orthologous"**. This additional table lists the names of genes found orthologous by the **Microbial Genome Database for Comparative Analysis **http://mbgd.genome.ad.jp/ Click here for file

Additional file 8 **"List of CDS in Mmc strains GM12 and 95010 showing less than 90% similarity with each other"**. This additional table lists the names of genes that are not highly similar in two strains belonging to the same subspecies, *M. mycoides *subsp. *capri*. Click here for file
