# Supplementary material for: Mycoplasma mycoides, from "mycoides Small Colony" to "capri". A microevolutionary perspective
Source: BMC Genomics. 2011 Feb 16;12:114. doi: 10.1186/1471-2164-12-114 (PMC3053259; doi:10.1186/1471-2164-12-114)
Supplement: Additional file 1 — "PCR primers used during this study". This additional table lists the names and sequences of primers used in the study. [file 1471-2164-12-114-S1.DOC]

Table S1. Primers used for PCR

| Name | Position | 5’-3’ sequence |
| --- | --- | --- |
| IR-IS*Mmy2* | 8-24 & 1351-1367^a^ | GGACAAAATTATTAGAC |
| IS*Mmy3*-F | 201673-201692 | CTGAATTTAAAATTGCTGTT |
| IS*Mmy3*-R | 200768-200787 | GTAATGAATTCCTTGATCAG |
| TraG-MLC-F | 275481-275504 | CAATGCTAAAAATGTTTTAGTTTG |
| TraG-MLC-R | 276004-276023 | GGAATTTCAGGAATTGATAA |
| VIP-F | 270115-270134 | AACGGACTAAAAGGTAAAGT |
| VIP-R | 380478-280497 | GCAGGAACTGCTACTAAATT |

Nucleotide positions on the Mmc 95010 sequence, except ^a^ positions in the ISMmy*2* sequence (Acc. N°: DQ887910)
