# Supplementary material for: Mycoplasma mycoides, from "mycoides Small Colony" to "capri". A microevolutionary perspective
Source: BMC Genomics. 2011 Feb 16;12:114. doi: 10.1186/1471-2164-12-114 (PMC3053259; doi:10.1186/1471-2164-12-114)
Supplement: Additional file 2 — "Comparison of related plasmid and ICE sequences in Mmc 95010". This figure describes the positions and sequences shared by the integrative conjugative elements copies and the plasmid that was characterized in the same strain. [file 1471-2164-12-114-S2.PPT]

## Slide 1
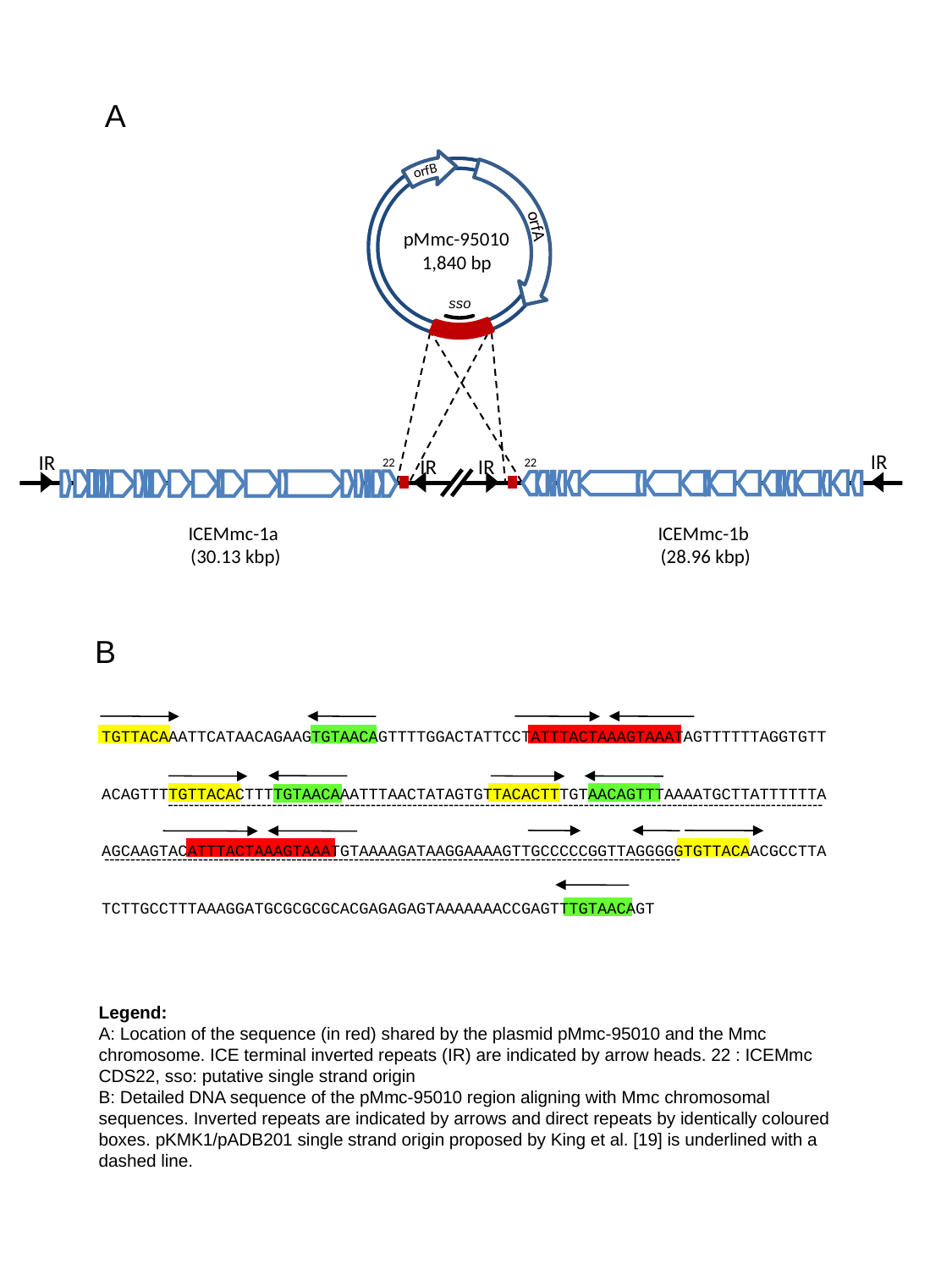

A
orfB
orfA
pMmc-95010
1,840 bp
IR
IR
IR
IR
22
22
ICEMmc-1a
(30.13 kbp)
ICEMmc-1b
(28.96 kbp)
sso
B
TGTTACAAATTCATAACAGAAGTGTAACAGTTTTGGACTATTCCTATTTACTAAAGTAAATAGTTTTTTAGGTGTT
ACAGTTTTGTTACACTTTTGTAACAAATTTAACTATAGTGTTACACTTTGTAACAGTTTAAAATGCTTATTTTTTA
AGCAAGTACATTTACTAAAGTAAATGTAAAAGATAAGGAAAAGTTGCCCCCGGTTAGGGGGTGTTACAACGCCTTA
TCTTGCCTTTAAAGGATGCGCGCGCACGAGAGAGTAAAAAAACCGAGTTTGTAACAGT
Legend:
A: Location of the sequence (in red) shared by the plasmid pMmc-95010 and the Mmc chromosome. ICE terminal inverted repeats (IR) are indicated by arrow heads. 22 : ICEMmc CDS22, sso: putative single strand origin
B: Detailed DNA sequence of the pMmc-95010 region aligning with Mmc chromosomal sequences. Inverted repeats are indicated by arrows and direct repeats by identically coloured boxes. pKMK1/pADB201 single strand origin proposed by King et al. [19] is underlined with a dashed line.
