# Supplementary material for: Mycoplasma mycoides, from "mycoides Small Colony" to "capri". A microevolutionary perspective
Source: BMC Genomics. 2011 Feb 16;12:114. doi: 10.1186/1471-2164-12-114 (PMC3053259; doi:10.1186/1471-2164-12-114)
Supplement: Additional file 3 — "Comparison of plasmids from the mycoides cluster". This figure depicts the results of a three-way BLASTN comparison of three mycoplasma plasmid sequences using the Artemis Comparison Tool [71] [file 1471-2164-12-114-S3.PPT]

## Slide 1
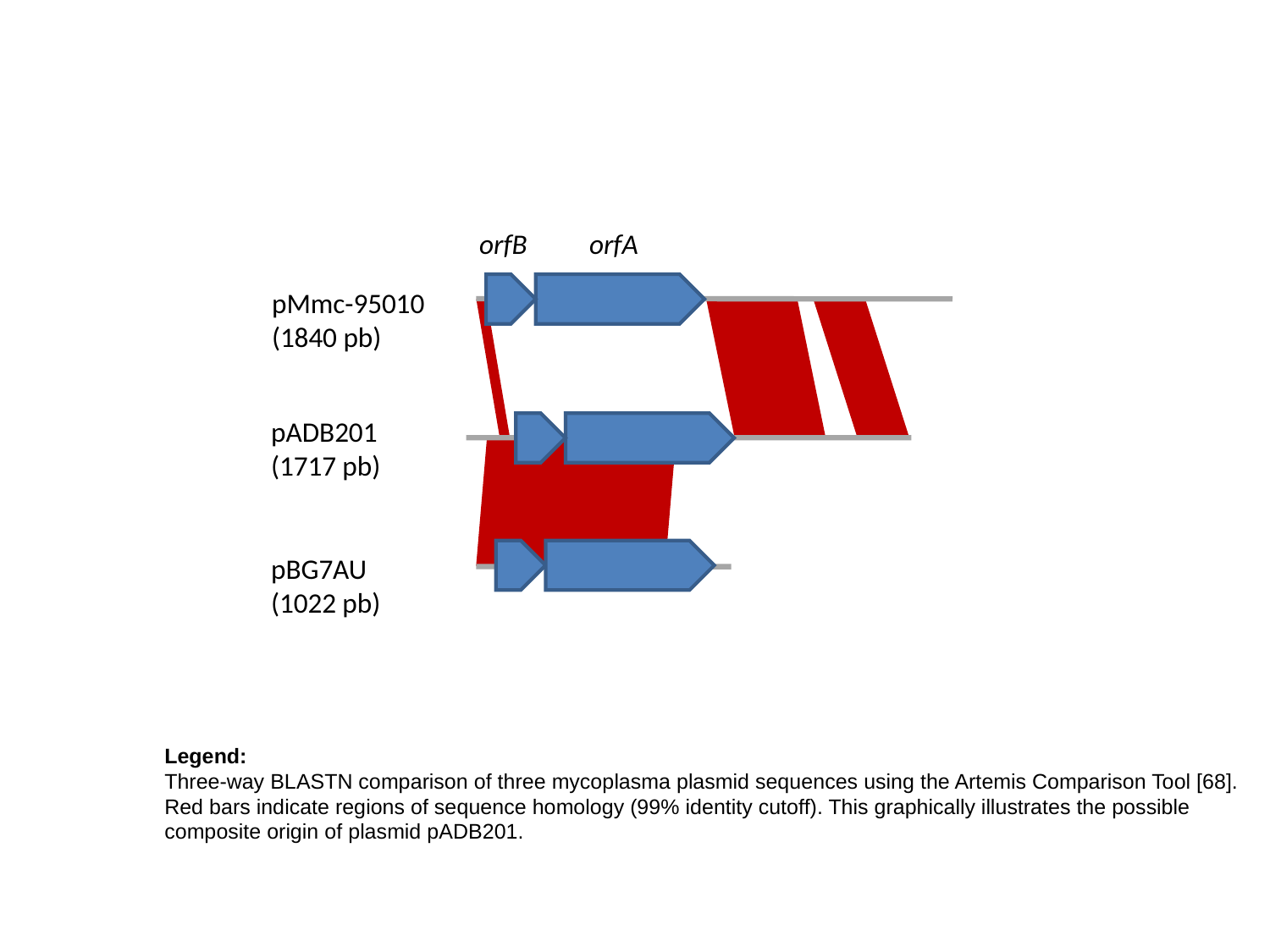

orfB
orfA
pMmc-95010
(1840 pb)
pADB201
(1717 pb)
pBG7AU
(1022 pb)
Legend:
Three-way BLASTN comparison of three mycoplasma plasmid sequences using the Artemis Comparison Tool [68]. Red bars indicate regions of sequence homology (99% identity cutoff). This graphically illustrates the possible composite origin of plasmid pADB201.
