# Supplementary material for: Mycoplasma mycoides, from "mycoides Small Colony" to "capri". A microevolutionary perspective
Source: BMC Genomics. 2011 Feb 16;12:114. doi: 10.1186/1471-2164-12-114 (PMC3053259; doi:10.1186/1471-2164-12-114)
Supplement: Additional file 4 — "Organization of lipoprotein gene clusters in the Mmc 95010 genome". This figure is a schematic representation of 6 lipoprotein families (A to F) found in the genome of strain 95010. [file 1471-2164-12-114-S4.PPT]

## Slide 1
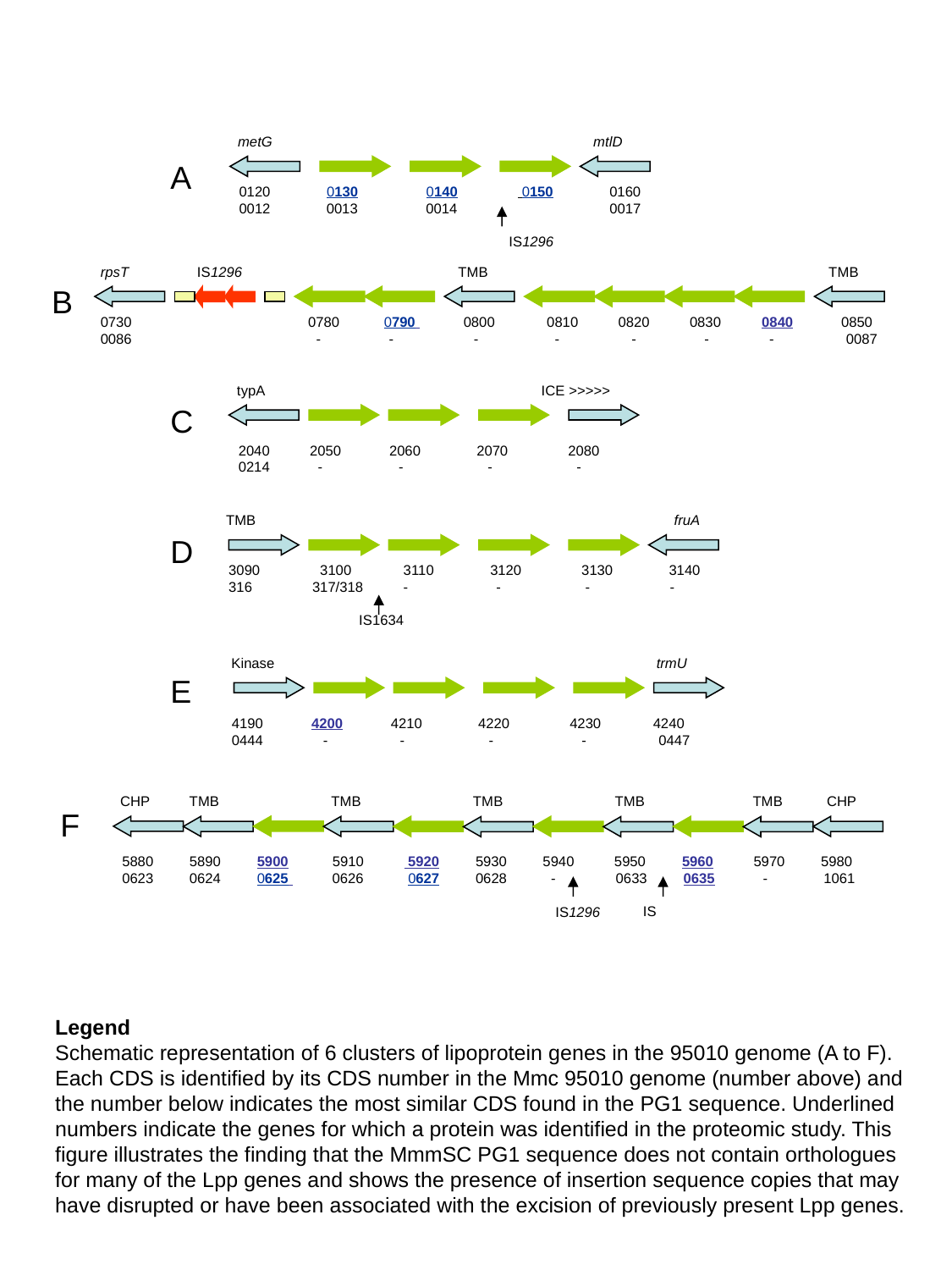

metG mtlD
0120 0130 0140 0150 0160
0012 0013 0014 0017
A
IS1296
rpsT IS1296 TMB TMB
B
0730 0780 0790 0800 0810 0820 0830 0840 0850
0086 - - - - - - - 0087
typA ICE >>>>>
2040 2050 2060 2070 2080
0214 - - - -
C
TMB fruA
3090 3100 3110 3120 3130 3140
316 317/318 - - - -
IS1634
D
Kinase trmU
4190 4200 4210 4220 4230 4240
0444 - - - - 0447
E
CHP TMB TMB TMB TMB TMB CHP
5880 5890 5900 5910 5920 5930 5940 5950 5960 5970 5980
0623 0624 0625 0626 0627 0628 - 0633 0635 - 1061
IS
IS1296
F
Legend
Schematic representation of 6 clusters of lipoprotein genes in the 95010 genome (A to F). Each CDS is identified by its CDS number in the Mmc 95010 genome (number above) and the number below indicates the most similar CDS found in the PG1 sequence. Underlined numbers indicate the genes for which a protein was identified in the proteomic study. This figure illustrates the finding that the MmmSC PG1 sequence does not contain orthologues for many of the Lpp genes and shows the presence of insertion sequence copies that may have disrupted or have been associated with the excision of previously present Lpp genes.
