# Supplementary material for: Mycoplasma mycoides, from "mycoides Small Colony" to "capri". A microevolutionary perspective
Source: BMC Genomics. 2011 Feb 16;12:114. doi: 10.1186/1471-2164-12-114 (PMC3053259; doi:10.1186/1471-2164-12-114)
Supplement: Additional file 5 — "organization of a Mmc-specific lipoprotein gene clusters in the Mmc 95010 genome". This figure is a schematic representation of a lipoprotein family found in strain 95010 genome but not in the published MmmSC genomes. [file 1471-2164-12-114-S5.PPT]

## Slide 1
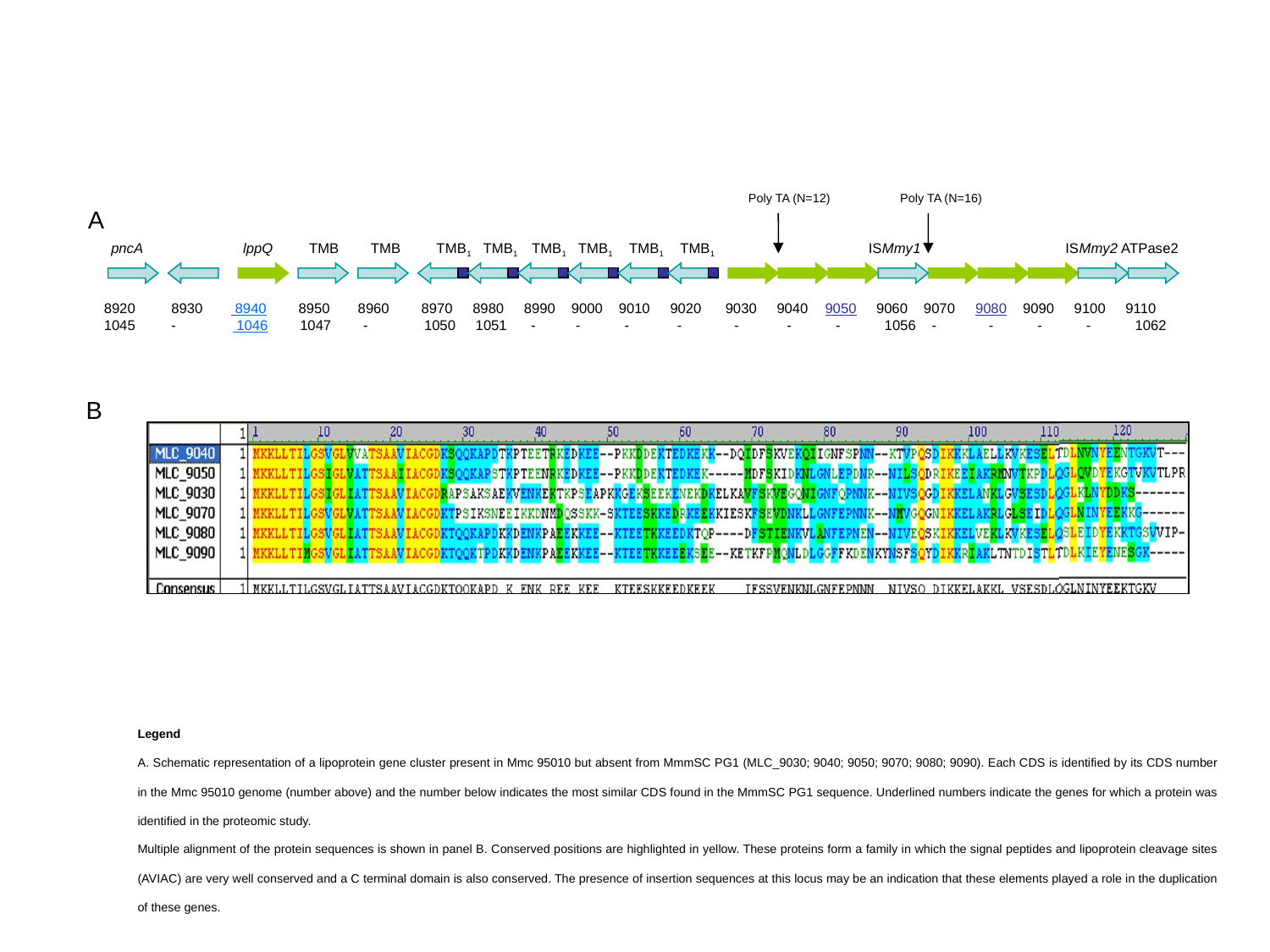

A
Poly TA (N=12)
Poly TA (N=16)
pncA lppQ TMB TMB TMB1 TMB1 TMB1 TMB1 TMB1 TMB1 ISMmy1 ISMmy2 ATPase2
8920 8930 8940 8950 8960 8970 8980 8990 9000 9010 9020 9030 9040 9050 9060 9070 9080 9090 9100 9110
1045 - 1046 1047 - 1050 1051 - - - - - - - 1056 - - - - 1062
B
Legend
A. Schematic representation of a lipoprotein gene cluster present in Mmc 95010 but absent from MmmSC PG1 (MLC_9030; 9040; 9050; 9070; 9080; 9090). Each CDS is identified by its CDS number in the Mmc 95010 genome (number above) and the number below indicates the most similar CDS found in the MmmSC PG1 sequence. Underlined numbers indicate the genes for which a protein was identified in the proteomic study.
Multiple alignment of the protein sequences is shown in panel B. Conserved positions are highlighted in yellow. These proteins form a family in which the signal peptides and lipoprotein cleavage sites (AVIAC) are very well conserved and a C terminal domain is also conserved. The presence of insertion sequences at this locus may be an indication that these elements played a role in the duplication of these genes.
