# Supplementary material for: Mycoplasma mycoides, from "mycoides Small Colony" to "capri". A microevolutionary perspective
Source: BMC Genomics. 2011 Feb 16;12:114. doi: 10.1186/1471-2164-12-114 (PMC3053259; doi:10.1186/1471-2164-12-114)
Supplement: Additional file 6 — "Peculiar Mmc genomic locus encoding predicted surface proteins". This figure is a schematic representation of the locus and homologies with M. agalactiae or M. capricolum subsp. capricolum genes. [file 1471-2164-12-114-S6.PPT]

## Slide 1
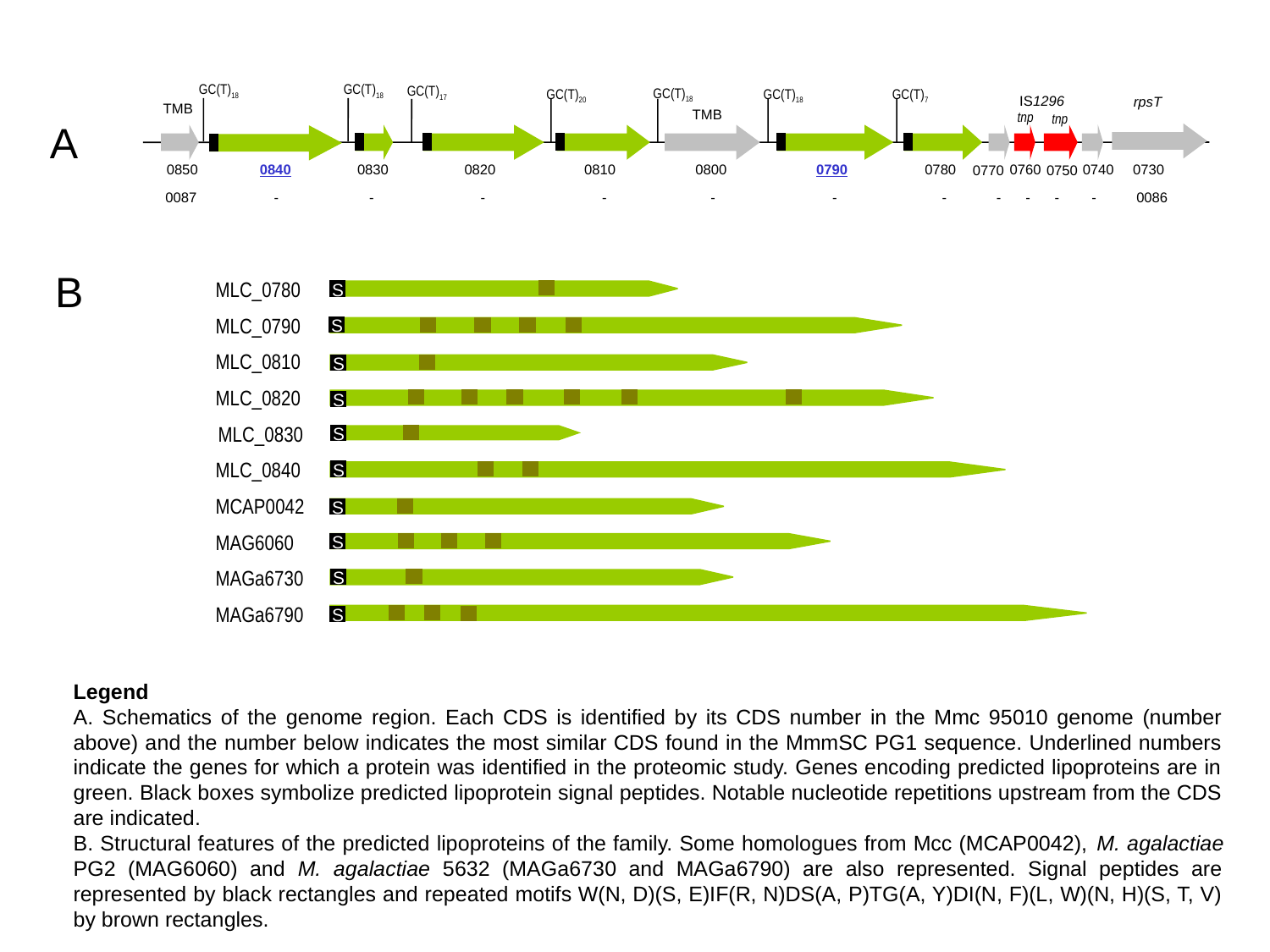

GC(T)18
GC(T)18
GC(T)17
GC(T)18
GC(T)20
GC(T)18
GC(T)7
IS1296
rpsT
TMB
TMB
tnp
tnp
A
0850
0840
0830
0820
0810
0800
0790
0780
0760
0740
0730
0770
0750
0087
-
-
-
-
-
-
-
-
-
-
-
0086
B
MLC_0780
S
MLC_0790
S
MLC_0810
S
MLC_0820
S
MLC_0830
S
MLC_0840
S
MCAP0042
S
MAG6060
S
MAGa6730
S
MAGa6790
S
Legend
A. Schematics of the genome region. Each CDS is identified by its CDS number in the Mmc 95010 genome (number above) and the number below indicates the most similar CDS found in the MmmSC PG1 sequence. Underlined numbers indicate the genes for which a protein was identified in the proteomic study. Genes encoding predicted lipoproteins are in green. Black boxes symbolize predicted lipoprotein signal peptides. Notable nucleotide repetitions upstream from the CDS are indicated.
B. Structural features of the predicted lipoproteins of the family. Some homologues from Mcc (MCAP0042), M. agalactiae PG2 (MAG6060) and M. agalactiae 5632 (MAGa6730 and MAGa6790) are also represented. Signal peptides are represented by black rectangles and repeated motifs W(N, D)(S, E)IF(R, N)DS(A, P)TG(A, Y)DI(N, F)(L, W)(N, H)(S, T, V) by brown rectangles.
